# Food for thought: the enhanced recall of metaphorical food sentences independent of hunger

**DOI:** 10.1007/s10339-024-01222-z

**Published:** 2024-08-30

**Authors:** Catherine Audrin, Géraldine Coppin

**Affiliations:** 1 University of Teacher Education, Lausanne, Switzerland; 2 Swiss Center for Affective Sciences, Geneva, Switzerland; 3 Present Address: Formation Universitaire à Distance (Unidistance), Brig, Switzerland

**Keywords:** Metaphor processing, Appraisal theories, Memory, Food, Hunger

## Abstract

Metaphorical sentences are assumed to be related to more costly processes than their literal counterparts. However, given their frequent use in our daily lives, metaphorical sentences “must come with a benefit” (Noveck et al. Metaphor Symb 16:109–121. 10.1080/10926488.2001.9678889, 2001). In this paper, we investigated whether metaphorical sentences were better remembered than their literal counterparts. In addition, we were interested in assessing whether the relevance of the metaphors impacted this recall. Anchoring this hypothesis in the appraisal theory, we hypothesized that food-related metaphorical sentences may be particularly relevant when one is hungry, and consequently, be better remembered in that particular physiological state. Participants were presented with randomized metaphorical sentences and their literal counterparts and were later asked to remember the missing word in both metaphorical and literal sentences. General mixed model analyses revealed that metaphorical sentences were better remembered. However, there was no significant effect of hunger. We discuss these results in relation to (1) the metaphor literature and (2) the appraisal theory of emotion.

## Introduction

For the past 30 years, metaphors have been studied in cognitive science (Way 1991). Metaphors are prevalent in natural language, making up as much as 20% of discourse. They can be instantiated by nouns (crime is a ‘virus’), verbs (crime ‘plagues’ the city), adjectives (‘infectious’ crime), and other parts of speech (Thibodeau et al. 2017). As emphasized by Thibodeau, metaphors consist of three components: a source domain, a target (or topic) domain, and a mapping between them. The source domain used in a metaphor (‘virus’) is more familiar than the target domain (‘crime’). Metaphors transfer aspects that normally apply to the first object onto the second object. Thus, metaphors help make sense of objects and give a specific meaning to the target domain (Sopory and Dillard 2002).

Two main lines of research on metaphors have emerged: Studies focusing on what metaphor productions reveal regarding people’s thoughts (Martínez et al. 2001), and studies investigating the impact of using metaphors to introduce a concept. The literature has emphasized the impact of metaphors on multiple aspects of behavior and cognition such as attitudes (Sopory and Dillard 2002), thoughts and reasoning (Thibodeau et al. 2017), behaviors (Gallagher et al. 2013), but also in learning, since metaphors can be used to teach new concepts (Aubusson et al. 2006) that may appear complex at first sight (Niebert and Gropengiesser 2013). However, how metaphors are understood has been debated (Holyoak and Stamenković 2018). Noveck et al. (2001) suggest that metaphorical understanding not only requires the individual to comprehend what the metaphor is referring to, but to also grasp the intentions behind the use of such a metaphor. This means that understanding metaphors should be more costly (i.e., require more processing time and deeper cognitive processing) than understanding their literal counterpart. The empirical evidence supporting this shows that children have more difficulty understanding metaphorical utterances (Vosniadou et al. 1984 in Noveck et al. 2001) and that adults take a longer time to read them than literal sentences (Gerring and Healy 1983 in Noveck et al. 2001). Noveck et al. replicated these results, and they found that both children and adults read metaphorical sentences more slowly than their literal counterparts. These results were further supported by Carston and Yan (2023), who showed across two experiments that metaphorical understanding was more costly (i.e., related to longer reading times) than literal understanding. Based on the evidence reported above, one could argue that the processing of metaphors is more costly and may occur at a deeper level, thus possibly enhancing their memorization. However, few studies have investigated the impact of metaphorical sentences on memory. One notable exception is the study of Craik and Tulving (1975), who revealed that metaphorical sentences embedded in stories were better remembered by college students than literal sentences– a different demographic than the present study. In addition, these metaphorical sentences increased the recall of the context preceding the metaphor.

Rather than assuming metaphor comprehension is constant across individuals and situations, the present study intends to investigate how specific aspects of metaphors and individuals may impact the recall of metaphors. More specifically, the purpose of this study was to assess whether the relevance of the metaphorical sentences has an impact on their recall. In this study, the metaphorical sentences relate to food. Food is a biologically relevant stimulus, as it is crucial for one’s survival. Evidence shows that hunger can modulate several cognitive processes (Benau et al. 2014), notably memory for food stimuli (Montagrin et al. 2019). Epstein and Levitt (1962) showed that learning and the recall of food-related words was increased by hunger. Since then, other studies have revealed that food items were better remembered than non-food items and that this was especially true when participants were hungry (Talmi et al. 2013; Montagrin et al. 2019). These results can be interpreted through the lens of appraisal theories. These theories posit that emotions arise from an evaluation process (Sander et al. 2005) triggered by the relevance of the stimulus encountered. More specifically, the “relevance hypothesis” postulates that when something is perceived as relevant for one’s goal, it not only induces an emotion but has a direct facilitatory effect on memory (Sander et al. 2005). In line with this hypothesis, studies have shown that once neutral items became goal relevant, they were better recalled (e.g., Montagrin et al. 2013, 2018).

This study investigates whether metaphorical sentences foster memory. More specifically, recall of missing words in metaphorical sentences related to food (“Die Idee ist total *Banane*”, this idea is totally “banana” - meaning stupid) was compared to the recall of missing words in their literal counterparts (“Die Idee ist total *doof*”, this idea is totally stupid). Given previous evidence and propositions from the appraisal theories, we first hypothesized that metaphorical food-related sentences should be better remembered than their literal non food-related counterparts. Second, we posited that this effect might be enhanced for hungry participants.

## Method

### Participants

One hundred and twenty-one participants (*M*_age_ = 36.06 ± 12.87 years) took part in this experiment (94 females). Most of the participants had completed a university degree – either a bachelor, master or doctoral degree (*n* = 67, 55.38%). The study was approved by the ethics committee of UniDistance Suisse’s Psychology Department. Participants were tested online and paid 40 Swiss Francs (approximately 40 US dollars) for their participation. Before starting the experiment, participants were asked to give their consent to participate in the study. Sample size estimation was based on Arend and Schäfer (2019)’s rules of thumb. Arend and Schäfer (2019) suggest that cross-level interaction effects can be detected from any combination between 200 participants with 9 items and 125 participants with 25 items at a power of 0.80. As 22 items were presented to each participant in each condition (see below), 125 participants were recruited but four of them did not give their consent for the use of their data. In the end, 121 participants took part in this study.

### Materials and procedure

Metaphorical sentences used in Citron and Zervos (2018) and in Citron and Goldberg (2014) were presented using Limesurvey. In this study, we kept 44 of the initial 74 items: 22 items in the metaphorical condition (e.g., “she gazed at him sweetly”) and 22 items in the literal condition (e.g., “she gazed at him cutely”, Table 1). Fewer items were presented so that only unique terms in the metaphorical sentences were kept – items such as “she looked at him sweetly” were too close to “she received a sweet compliment”. As such, only one of these items was kept. All the sentences were presented in German and were highly conventional German expressions. The sentences were matched for their emotional valence, emotional arousal, imageability, number of words and number of letters (Citron and Zervos 2018; Citron and Goldberg 2014).

**Table 1:** List of the stimuli by type of condition (metaphorical / literal) – reported with permission from Citron and colleagues (2014)

Type	Original German sentences	English translations
Metaphorical	Sie blickte ihn *süß* an	She gazed at him *sweetly*
Literal	Sie blickte ihn *niedlich* an	She gazed at him *cutely*
Metaphorical	Er hat ein *zuckersüßes* Lächeln	He has a *sugar-sweet* smile
Literal	Er hat ein *charmantes* Lächeln	He has a *charming* smile
Metaphorical	Er war *sauer* auf sie	He was *sour* at her
Literal	Er war *wütend* auf sie	He was *angry* at her
Metaphorical	Er schaut immer so *verbittert* drein	He always looks so *bitterly* into it
Literal	Er schaut immer so *enttäuscht* drein	He always looks so *disappointingly* into it
Metaphorical	Bei den Wahlen kam es zu *herben* Verlusten	At the elections, it came to *acetous* losses
Literal	Bei den Wahlen kam es zu *großen* Verlusten	At the elections, it came to *big* losses
Metaphorical	Der Abbruch war sehr *bitter* für ihn	The breakup was very *bitter* for him
Literal	Der Abbruch war sehr *schlimm* für ihn	The breakup was very *bad* for him
Metaphorical	Sie bekamen eine *gesalzene* Rechnung	They received a *salty* bill
Literal	Sie bekamen eine *hohe* Rechnung	They received a *high* bill
Metaphorical	Diese Boutique hat *gepfefferte* Preise	This boutique has *peppered* prices
Literal	Diese Boutique hat *hohe* Preise	This boutique has *high* prices
Metaphorical	Sie war sehr *scharf* auf ihn	She was very *spicy* toward him
Literal	Sie war sehr *angetan* von ihm	She was very *keen* on him
Metaphorical	Der Fall ist diplomatisch äußerst *pikant*	The case is diplomatically extremely *spicy*
Literal	Der Fall ist diplomatisch äußerst *heikel*	The case is diplomatically extremely *delicate/awkward*
Metaphorical	Das Kind bekam eine *saftige* Ohrfeige	The child got a *juicy* slap
Literal	Das Kind bekam eine *starke* Ohrfeige	The child got a *strong* slap
Metaphorical	Ihre Äußerungen sind wirklich *geschmacklos*	Her statements are really *tasteless*
Literal	Ihre Äußerungen sind wirklich *unverschämt*	Her statements are really *impertinent*
Metaphorical	Die Bilder waren *unappetitlich*	The pictures were *unappetizing*
Literal	Die Bilder waren *verstörend*	The pictures were *disturbing*
Metaphorical	Sie ist ein *leckeres* Mädchen	She is a *tasty* girl
Literal	Sie ist ein *hübsches* Mädchen	She is a *beautiful* girl
Metaphorical	Hoffentlich treffen wir ihren *Geschmack*	Hopefully we meet her *taste*
Literal	Hoffentlich treffen wir ihren *Stil*	Hopefully we meet her *style*
Metaphorical	Der Fernsehbericht hat ordentlich *Würze*	The TV report has proper *seasoning*
Literal	Der Fernsehbericht ist sehr *reizvoll*	The TV report is of great *interest*
Metaphorical	Dieser Mann ist ein echter *Leckerbissen*	This man is a really *tasty bite*
Literal	Dieser Mann ist echt *hübsch*	This man is really *handsome*
Metaphorical	Dieses Kleid ist ein wahrer *Augenschmaus*	This dress is a real *eye-feast* (i.e., delicacy) for the eyes)
Literal	Dieses Kleid ist wahrhaft *schön*	This dress is a real *beauty*
Metaphorical	Sie ist eine echte *Sahneschnitte*	She is a real *cream slice*
Literal	Sie ist eine echt *attraktive Frau*	She is a really *attractive woman*
Metaphorical	Die Idee ist total *Banane*	This idea is completely *banana*
Literal	Die Idee ist total *doof*	This idea is completely *dumb*
Metaphorical	Was sie erzählt ist echt *Quark*	What she is saying is really *quark*
Literal	Was sie erzählt ist echt *Quatsch*	What she is telling is really *balderdash*
Metaphorical	Dieser Zeitungsartikel ist totaler *Käse*	This newspaper article is a total *cheese*
Literal	Dieser Zeitungsartikel ist totaler *Unsinn*	This newspaper article is totally *nonsense*

**Fig. 1:**
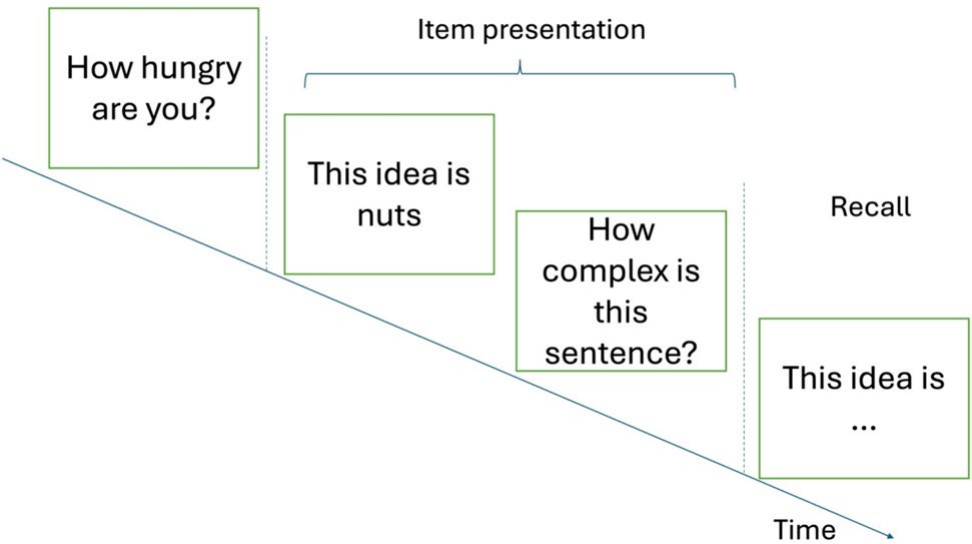
Experimental procedure

The experiment was conducted as described in Fig. 1. For each step of the experiment, participants were free to take as much time as they needed. First, participants were asked to answer demographic questions regarding their gender, age, and level of education. Then, they were asked to rate how hungry they were when they started the experiment on a scale from 1 (*not hungry at all*) to 7 (*extremely hungry*). To keep the experiment engaging, participants were asked to assess the sentences after their presentation (such as “how complex is this sentence?”). However, an analysis of these answers is beyond the scope of this paper. In a second phase that was unexpected for the participants, they were asked to remember the missing word in both metaphorical and literal sentences. For example, they were presented with “Die Idee ist total …” (“The idea is totally….”) and were asked to freely recall the missing word.

The experiment was conducted in German. All materials are freely available upon request.

### Data analysis

Analyses were performed using R version 12.1 (R Development Core Team 2008), lmerTest (Kuznetsova et al. 2017) and lme4 packages (Bates et al. 2015).

Citron and Goldberg’s ratings were used in Analyses of Variance (ANOVAs) to determine whether the metaphorical and literal items kept in this study differed in terms of their (1) familiarity, (2) metaphoricity, (3) imageability, (4) taste-reference, (5) emotional valence and (6) emotional arousal.

The recall variable is a dichotomous measure of the participants’ accuracy in recalling the missing word. If the item was correctly recalled, it was coded 1, and if it was left blank or incorrectly recalled, it was coded 0. The following analyses were performed on the recall variable. First, descriptive analyses were performed on the percentage of words recalled in each experimental condition (metaphorical or literal). Then, generalized linear mixed model (GLMM) analyses were conducted on the recall variable (0/1). Participants and stimuli were introduced as random intercepts, and the condition was introduced as a random slope for participants and items. The latter allowed us to distinguish the variance related to the stimuli versus the experimental condition. Condition (metaphorical vs. literal) was integrated as a fixed effect, along with the participants’ level of hunger. The following effects were tested: the main effect of condition, the main effect of hunger as well as the interaction between condition and hunger.

## Results

### Preliminary analyses

Preliminary analyses contrasting metaphorical and literal items regarding their (1) familiarity, (2) metaphoricity, (3) imageability, (4) taste-reference, (5) emotional valence, and (6) emotional arousal revealed that metaphorical sentences were significantly higher than literal sentences in terms of taste-relatedness (*F*(1,21) = 150, *p* < .001), and metaphoricity (*F*(1,21) = 107, *p* < .001). As for familiarity, metaphorical sentences were significantly less familiar than literal sentences (*F*(1,21) = 11.3, *p* < .001). However, there were no significant differences between the metaphorical and literal sentences for imageability (*F*(1,21) = 0.05, *p* = .822), emotional valence (*F*(1,21) = 1.89, *p* = .183), emotional arousal (*F*(1,21) = 3.45, *p* = .078), and number of words (*F*(1,21) = 1, *p* = .328). There was however a significant difference in number of letters (*F*(1,21) = 4.99, *p* = .036), metaphorical sentences had significantly more letters than the literal ones (*M*_metaphorical sentences_ = 34.0; *SD* = 6.38; *M*_literal sentences_ = 32.2, *SD* = 5.78).

### Analyses on the recall variable

Descriptive analyses (percentage of correct answers for each condition) are reported in Table 2.

**Table 2:** Descriptive statistics of the percent of correct answers by condition (literal vs. metaphorical)

	Literal	Metaphorical
Incorrect (0)	75.24	37.94
Correct (1)	24.75	62.05

**Fig. 2:**
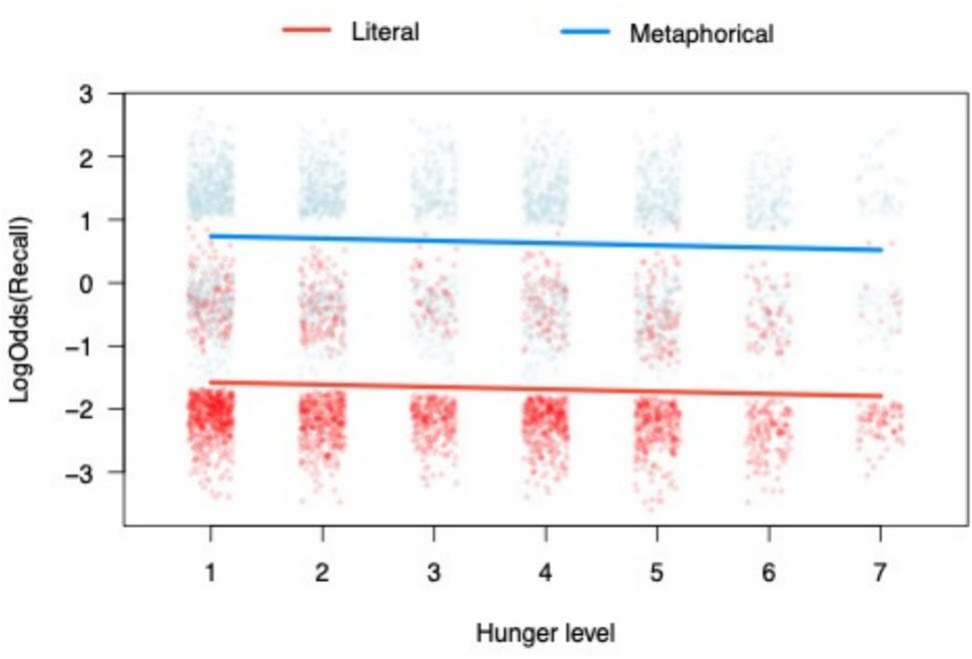
Graphical representation of the main effect of metaphorical sentences on recall

The results of the analyses performed to test the effect of condition and hunger on recall are depicted in Fig. 2 and reported in Table 3. These revealed a large significant effect of condition (*OR* = 0.30, *95%CI* = [0.23;0.40], *z* = 8.482, *p* < .001, *R*^*2*^_*marginal*_ with the condition effect = 0.196); metaphorical sentences were better recalled than their literal counterparts. Contrary to the hypotheses, results revealed no main effect of hunger (OR = 0.97, *95%CI* = [0.88;1.07], *z* = 0.54, *p* = .589), nor any interaction between hunger and condition (*OR* = 1.01, *95%CI* = [0.95;1.08], *z* = 0.41, *p* = .678).

**Table 3:** Summary of the mixed-effects model analyses predicting recall as a function of condition (literal vs. metaphorical) and level of hunger

	Recall (0/1)				
Predictors	Odds ratio	SD	CI	z	*p*
Intercept	0.66	0.28	0.38–1.15	− 1.47	0.143
Condition	0.30	0.14	0.23–0.40	− 8.48	**< 0.001**
Hunger	0.97	0.05	0.88–1.07	− 0.54	0.589
Condition*Hunger	1.01	0.03	0.95–1.08	0.41	0.678

Random effects		
Participants	Variance	SD
Intercept	0.77	0.88
Slope	0.29	0.54
*Items*		
Intercept	1.06	1.03
Slope	0.10	0.32
Marginal R^2^ / Conditional R^2^	0.196 / 0.520	

Bold value represents significant effect

## Discussion

The purpose of this paper was to investigate whether metaphorical sentences were better remembered than their literal counterparts. While the understanding of metaphors has been extensively investigated in the literature, few studies have focused on metaphor recall. Based on previous evidence and the appraisals theory, we hypothesized that (i) metaphorical sentences related to food are better remembered than literal sentences, and (ii) hunger is associated with a better recall of food-related metaphorical sentences.

Consistent with the first hypothesis, metaphorical sentences were better remembered even though metaphorical words had significantly more letters than the literal ones. Interestingly, the impact of metaphorical sentences appear to be strong enough to overcome the impact of word length (Baddeley et al. 1975). This result is line with Reynolds and Schwartz’s (1983) findings. In their study, they presented metaphorical (vs. literal) sentences as the final sentence of passages participants read. Their results revealed that metaphors as well as the rest of the passages preceding the final sentence were better remembered than the literal sentences and context. Our results build on previous evidence contrasting metaphorical and literal sentences. Consistent with Reynolds and Schwartz (1983), our results suggest that metaphors may enhance recall. However, as noted earlier, there are few studies specifically investigating metaphor recall. Nonetheless, the literature has extensively explored how metaphors are understood and processed.

Previous evidence has revealed that metaphors were (1) less understood (Noveck et al. 2001) and (2) related to longer reading times (Carston and Yan 2023). The present study highlights that metaphor processing was related to enhanced memory, which may suggest that metaphor processing is a costly task (Noveck et al. 2001; Carston and Yan 2023). Interestingly, the better recall of metaphorical sentences was strong and significant despite metaphorical words being significantly longer than literal words. This may be related to the benefits mentioned by Noveck et al. (2001) related to metaphor processing. In their paper, they notably argue that “the extra costs associated with an apt metaphor should come with benefits” (Noveck et al. 2001, p. 109) – better recall may be a strong indicator of such a benefit.

One potential explanation for the better recall of metaphorical sentences may be that metaphor processing is related to deeper processing (Craik and Tulving 1975). While this was not tested in this study, Mon et al. (2021) specifically investigated whether metaphors were more engaging than their literal counterparts. They highlighted that while metaphors and their literal counterparts were judged to have a similar meaning, metaphors elicited greater pupil dilation than literal sentences. The authors proposed pupil dilation occurs when participants show enhanced attention or higher task engagement. This suggests that metaphors are processed at a deeper level. This is also concordant with previous neuroimaging results, which revealed that metaphors were more engaging than their literal counterparts (Citron et al. 2019; Citron and Goldberg 2014). In their results, like in our study, the observed effect could not be due to enhanced difficulty or effort as the literal and metaphorical stimuli were controlled for familiarity and complexity. In addition, Mon et al. (2021) further investigated if their findings could have been driven by the fact that metaphors were perceived as more emotional. In their last study, they asked participants to assess whether they thought a metaphor (or its literal counterpart) expressed (1) more emotion, (2) more information, or (3) had a richer meaning. They found that a richer meaning was the most important difference between metaphors and literal sentences, suggesting that metaphors were not perceived as more emotional than their literal counterparts. Similarly, participants in the present study found the emotional valence and arousal to be comparable between metaphors and literal stimuli. These results are in contradiction with previous evidence suggesting that enhanced amygdala activation when processing metaphors reflected more emotional engagement (Citron and Goldberg 2014), as well as with other behavioral and neuroimaging studies suggesting that metaphors are related to more emotional involvement (Audrin and Coppin 2022; Aziz-Zadeh and Gamez-Djokic 2016; Mohammad et al. 2016). Further investigation is necessary due to the inconclusive results among the literature (Pomp et al. 2018). However, appraisal theories may provide interesting insights in understanding the potential emotionality related to metaphors. These theories emphasize that emotions arise from an evaluative process, starting with assessment of the relevance of the stimulus encountered. Once the stimulus is perceived as relevant, emotions are triggered. In this instance, however, the hypothesis investigating the impact of the relevance of metaphorical sentences on recall was not supported; no evidence was found regarding the impact of hunger (relevance) on recall (nor an interaction with the experimental condition). There could be several explanations for this result. First, most of our participants (45.54%) were satiated, and only a small fraction (3%) was hungry. Future experiments could systematically manipulate levels of hunger and food intake (DiFeliceantonio et al. 2018) prior to the task to test the impact of hunger on food-related metaphorical sentences. Second, the metaphorical sentences used contained terms such as “sweet”, “sour”, “bitter”, “acetous” and “peppered”. These terms are mostly descriptive of food-related perceptions, but only loosely related to actual food items or food consumption. Third, the meaning of the taste words was abstract (Citron and Goldberg 2014). The extent to which hunger could modulate the memory for such an abstract meaning could consequently be dampened.

Despite these limitations, this study offers valuable insights into the ongoing debate about metaphor processing. Our findings provide additional evidence to existing research, showing that metaphorical sentences are better recalled, which may indicate deeper processing of these sentences. However, further research is needed to clarify the mechanisms underlying the comprehension of metaphors.

## Conclusion

The current findings showed that metaphorical sentences are better remembered than their literal counterparts. This finding on metaphors is interpreted through the lens of previous research investigating the costs and benefits related to metaphorical processing (Noveck et al. 2001; Carston and Yan 2023). We draw conclusions on the impact of hunger on the memory for food-related metaphorical sentences, but we encourage further investigations to study this aspect.

## Data Availability

The original data are available upon request from the authors. CA and GC designed the study. CA programmed the experiment and ran the analyses. CA and GC interpreted the data and wrote the manuscript.
